# Marine picoplankton metagenomes and MAGs from eleven vertical profiles obtained by the Malaspina Expedition

**DOI:** 10.1038/s41597-024-02974-1

**Published:** 2024-02-01

**Authors:** Pablo Sánchez, Felipe H. Coutinho, Marta Sebastián, Massimo C. Pernice, Raquel Rodríguez-Martínez, Guillem Salazar, Francisco Miguel Cornejo-Castillo, Stéphane Pesant, Xabier López-Alforja, Ester María López-García, Susana Agustí, Takashi Gojobori, Ramiro Logares, Maria Montserrat Sala, Dolors Vaqué, Ramon Massana, Carlos M. Duarte, Silvia G. Acinas, Josep M. Gasol

**Affiliations:** 1 Institut de Ciències del Mar, CSIC, Passeig Marítim de la Barceloneta 37-49, 08003 Barcelona, Spain; 2 Departamento de Biotecnología, Facultad de Ciencias del Mar y Recursos Biológicos, Universidad de Antofagasta, Antofagasta, Chile; 3 Laboratorio de Complejidad Microbiana y Ecología Funcional, Instituto Antofagasta, Universidad de Antofagasta, Antofagasta, Chile; 4 Centre for Biotechnology & Bioengineering (CeBiB), Santiago, Chile; 5 Institute of Microbiology and Swiss Institute of Bioinformatics, ETH Zürich, Zürich, Switzerland; 6 EMBL’s European Bioinformatics Institute (EMBL-EBI), Hinxton, UK; 7 Centre National de la Recherche Scientifique (CNRS), UMR5254, IPREM, Pau, France; 8 King Abdullah University of Science and Technology (KAUST), Red Sea Research Center (RSRC) and Computational Bioscience Research Center (CBRC), Thuwal, Saudi Arabia

**Keywords:** Marine biology, Microbial ecology, Metagenomics

## Abstract

The Ocean microbiome has a crucial role in Earth’s biogeochemical cycles. During the last decade, global cruises such as *Tara* Oceans and the Malaspina Expedition have expanded our understanding of the diversity and genetic repertoire of marine microbes. Nevertheless, there are still knowledge gaps regarding their diversity patterns throughout depth gradients ranging from the surface to the deep ocean. Here we present a dataset of 76 microbial metagenomes (MProfile) of the picoplankton size fraction (0.2–3.0 µm) collected in 11 vertical profiles covering contrasting ocean regions sampled during the Malaspina Expedition circumnavigation (7 depths, from surface to 4,000 m deep). The MProfile dataset produced 1.66 Tbp of raw DNA sequences from which we derived: 17.4 million genes clustered at 95% sequence similarity (M-GeneDB-VP), 2,672 metagenome-assembled genomes (MAGs) of Archaea and Bacteria (Malaspina-VP-MAGs), and over 100,000 viral genomic sequences. This dataset will be a valuable resource for exploring the functional and taxonomic connectivity between the photic and bathypelagic tropical and sub-tropical ocean, while increasing our general knowledge of the Ocean microbiome.

## Background & Summary

The ocean is the largest biome on Earth. Microorganisms, mainly bacteria and archaea^1^ make up the majority of marine biomass and biodiversity and play a crucial role in biogeochemical cycles^2^. After pioneering work in the GOS expedition^3^, the main worldwide exploration of the marine microbiome through the analyses of microbial metagenomes have been those of the *Tara* Oceans Expedition (2009–2013)^4^, the Malaspina 2010 Expedition^5^, and more recently the Bio-GEOTRACES^6^ and Bio-GO-SHIP programs^7^.

Specifically, the Malaspina 2010 Circumnavigation Expedition^5^ sampled the marine microbiome in tropical and sub-tropical oceans, from the surface down to bathypelagic waters (~4,000 m depth) between 2010 and 2011. This emphasis on the vertical dimension by providing data that can be used to address geographical variation, is complementary to initiatives such as the Hawaii Ocean Time-Series^8^ which provides datasets that can be used to address temporal variation, both of which are fundamental for elucidating diversity variation patterns across the sunlit and dark oceans. In the photic ocean, the analyses of prokaryotic 16S rRNA gene tags, hereafter 16S TAGs metabarcoding datasets pointed to shifts towards communities enriched in rare taxa reflecting environmental transitions^9^ and both 16S and 18 S TAGs metabarcoding highlighted the role of dispersion on planktonic and micro-nektonic organisms^10^. In the dark ocean, the Malaspina Expedition contributed with an assessment of the diversity and biogeography of deep-sea pelagic prokaryotes^11^ as well as that of heterotrophic protists, unveiling the special relevance of fungal taxa^12^.

It also shed light on the ecological processes driving the diversity of free-living and also particle-attached bathypelagic prokaryotes, of which the latter had been historically overlooked, showing that particle-association lifestyle is a phylogenetically conserved trait in the deep ocean^13^.

Additionally, the first 58 microbial metagenomes of the bathypelagic ocean allowed us to reconstruct 317 high-quality metagenome-assembled genomes to metabolically characterize the deep ocean microbiome^14^, and also revealed that viruses reconstructed from particle-attached and free-living microbial cellular metagenomes exhibited contrasted diversity and auxiliary metabolic gene content^15^. Here, we present: 1) a new metagenomic resource of the ocean picoplankton (0.22 to 3.0 µm size fraction), formed by eleven detailed vertical profiles, from surface photic layers down to 4,000 m deep, covering the DCM, the mesopelagic and the bathypelagic realm with 3–4 sampling depths. Therefore, the Malaspina Microbial Vertical Profiles metagenomes dataset (MProfile) complements previous metagenomic data sets derived from the *Tara* Oceans expedition that sampled from surface waters through the mesopelagic ocean and our previous Malaspina bathypelagic deep ocean metagenomes dataset^14^, and 2) the new Malaspina Vertical Profiles metagenome-assembled genomes (MAGs) dataset with a total of 2,672 medium and high-quality MAGs of Archaea and Bacteria (Malaspina-VP-MAGs), and over 100,000 viral genomic sequences.

**Fig. 1:**
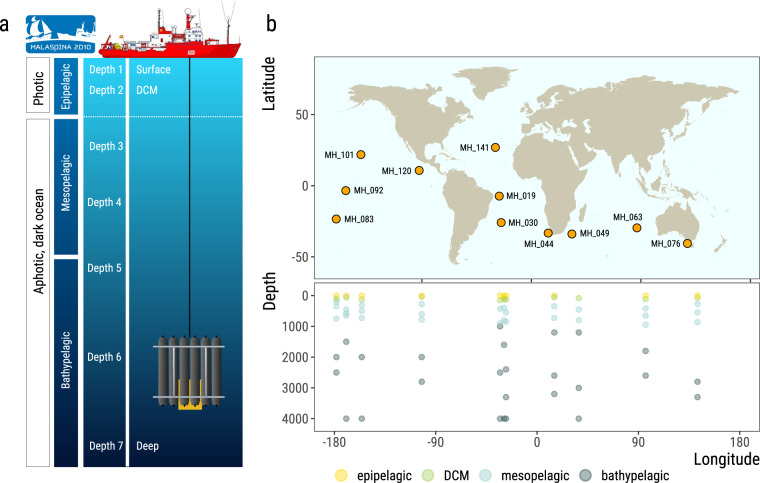
The Malaspina expedition microbial picoplankton vertical profiles. (**a**) Schematics of a typical vertical profile sampling event. Water samples were collected at seven depths from the surface to the ocean bottom or 4,000 m deep, targeting 3 layers from the photic and dark ocean: epipelagic, including surface and DCM, mesopelagic and bathypelagic. **(b)** Map showing the sampling stations of the Malaspina Expedition presented in this data set, along the tropical and sub-tropical global Ocean, and the depths from where water was collected for metagenomic sequencing of the 0.2–3 µm plankton size fraction.
(ii) a total of 25.3 Gbp of assembled contigs (332.9 Mbp ± 50.3 per sample, mean ± standard deviation obtained following the bioinformatics workflow depicted in Fig. 2; Supplementary Table 4). A fraction of 67.1% ± 1.8 of the predicted coding DNA sequences (CDS) in the assembled contigs (446,287 ± 63,375) could be assigned to at least one functional category (Fig. 3): 32.7% ± 1.5 to clusters of orthologous groups (COG)^16^, 63.7% ± 1.6 to protein families (PFAM)^17^, 28.3% ± 1.8 to Kyoto Encyclopedia of Genes and Genomes (KEGG)^18^ orthologs (KO), and 1.1% ± 0.1 to carbohydrate-active enzymes (CAZy)^19^. A fraction of 34.7% ± 1.6 of CDS could not be assigned to a function with the used databases (Supplementary Table 4). Similarly, 41.7% ± 10.7 CDS (187,334 ± 42,691) per sample could not be taxonomically classified further than “root” or were “unclassified” after aligning them to UniRef90^20^ using the lowest common ancestor approach (LCA).
(iii) a 17.43 million non-redundant CDS database (M-GeneDB-VP). In total 9,967,787 (57.2%) genes in this gene catalog were annotated with PFAM, 3,717,395 (21.3%) with KOs, 5,097,211 (29.3%) with COGs and 169,855 (0.97%) with CAZy, whereas 8,889,665 genes (43.1%) could not be annotated and correspond to the gene novelty of this database.
(iv) functional profiles of each gene grouped by annotation, consisting of the abundance of each CDS/annotation per sample, based on the number of reads of each metagenome mapping back to the M-GeneDB-VP, corrected by gene length and by single-copy universal marker gene abundance (see below).
(v) A 2,672 medium and high-quality Metagenome-Assembled Genomes (MAGs) of bacteria and archaea (Malaspina-VP-MAGs) with their functional and taxonomic annotations, corresponding to microorganisms from 22 bacterial and 5 archaeal phyla.
(vi) taxonomic profiles of picoplankton, based on the 16S mTAG^21^ analysis of the metagenomes, including 15,046 OTUs (Fig. 4).
(vii) A total of 101,219 viral genomic sequences of at least one kbp identified in the assembled contigs and 3,105 unique viral sequences identified as prophages within the MAGs sequences.

This resource consists on:

(i) primary data in the form of 1.66 Tbp of environmental whole genome shotgun sequencing data (Illumina 2 × 101 pair-end reads), distributed over 76 samples (Fig. 1) corresponding to 7 depths in 11 vertical profiles (108.1 ± 2.8 million read pairs, mean ± sd, and 21.8 ± 0.6 Gbp per sample), collected along the track of R/V Hespérides across tropical and sub-tropical regions of the global ocean during the Malaspina Expedition in 2010–2011.

This Malaspina Microbial Vertical Profiles metagenomes dataset (MProfile) resource will be of great interest to the community to tackle ecologically relevant questions on marine microbial ecology –recently it has been used to identify a universal scaling relationship between prokaryotic genome size and ocean temperature^22^– such as inferring differential functional traits of photic and aphotic bacterial and archaeal genomes through the reconstruction of metagenome assembled genomes (MAGs), as well as serve as a valuable dataset for gene discovery, with interest in biotechnology and other research areas.

Primary sequencing data and Megahit assemblies have been submitted to the European Nucleotide Archive. Derived data such as M-GeneDB-VP, annotations and files for functional and taxonomic profiles, MAG sequences, MAGs descriptive information, MAGs annotation, MAGs coding DNA and protein encoding gene sequences, viral genomic sequences, viral genomic sequences descriptive information and annotation of virus-derived coding DNA sequences have been submitted to the European Bioinformatics Institute BioStudies repository (accession S-BSST1059^23^) to allow further exploration of the functional and taxonomic composition and vertical connectivity of the ocean microbiome.

## Methods

### Sample collection

A total of 76 water samples were taken during the Malaspina 2010 expedition (http://www.expedicionmalaspina.es) on board the R/V Hespérides from January 4 to July 5, 2011 (Supplementary Table 2), corresponding to 11 different sampling stations (Fig. 1b). Each station was profiled by collecting water from 7 discrete depths (except station MH_120, with 6 depths) from surface (3 m) to the bathypelagic layer, down to 4,000 m (mean maximum depth of each profile 3,491 ± 626 m), including the deep chlorophyll maximum (DCM; Fig. 1b; Supplementary Table 2). Water samples were collected either with a rosette of Niskin bottles (12 L each) on a frame with a CTD sensor or with a large Niskin bottle (30 L) for the surface samples. For every sample, two 6-L replicates were pre-filtered sequentially through 200 µm and 20 µm nylon meshes to remove large plankton, and then through a 47 mm diameter polycarbonate (PC) membrane with a 3 µm pore size (Whatman filter ref: 10418312), and a 47 mm diameter PC membrane with a 0.22 µm pore size (Whatman filter ref: GTTP04700) using a peristaltic pump (Masterflex, EW-7741010) with a flow rate of 50–100 ml min^−1^. When the filtration rate decreased considerably, filters were replaced. The 0.22 µm filters, including the free-living (FL) prokaryotic community^24,25^ as well as picoeukaryotes, were packaged in 2-mL cryotubes, flash frozen in liquid nitrogen and stored in a freezer at −80 °C. All collection equipment was decontaminated between samples using ethanol and 0.1% bleach. The time span from bottle closing of the deep sample to filter freezing was approximately 4 h, and except for the time needed to empty the rosette bottles, the water was kept at 4 °C.

### DNA extraction

DNA was extracted with the standard phenol-chloroform protocol with slight modifications^21,26^. Detailed description of the DNA extraction protocol used in our lab have been previously published^27^. Briefly, the filters were cut in small pieces with sterile razor blades and resuspended in 3 mL of lysis buffer (40 mM EDTA, 50 mM Tris-HCl, 0.75 M sucrose). Samples were incubated at 37 °C for 45 min in lysis buffer (Lysozyme; 1 mg mL^−1^ final concentration) with gentle agitation. Then, the buffer was supplemented with sodium dodecyl sulfate (SDS, 1% final concentration) and proteinase K (0.2 mg mL^−1^ final concentration) and the samples were incubated at 55 °C for 60 min under gentle agitation. The lysate was collected and processed with the standard phenol-chloroform extraction procedure: an equal volume of Phenol:CHCl_3_:IAA (25:24:1, vol:vol:vol) was added to the lysate, mixed and centrifuged 10 min at 3,000 rpm. Then the aqueous phase was recovered and the procedure was repeated. Finally, residual phenol was removed by adding an equal volume of CHCl_3_:IAA (24:1, vol:vol) to the recovered aqueous phase. The mixture was centrifuged and the aqueous phase was recovered for further purification. The aqueous phase was then concentrated by centrifugation with a Centricon concentrator (Millipore, Amicon Ultra-4 Centrifugal Filter Unit with Ultracel-100 membrane). This step was repeated three times by adding 2 mL of sterile Milli-Q water each time to wash away any impurities that could interfere with the library preparation. The genomic DNA extract was concentrated down to 100 to 200 μL of volume.

### Sequencing

An average of 0.6 µg (minimum of 0.25 µg) of extracted DNA was sent and sequenced on the Illumina HiSeq 2000 platform at the Centre Nacional d’Anàlisi Genòmica (CNAG) in Barcelona, Spain. The libraries were sequenced using TruSeq SBS Kit v3-HS (Illumina, Inc), in paired-end mode with a read length of 2 × 101 bp following the manufacturer’s protocol. Images analysis, base calling and quality scoring of the run were processed using the manufacturer’s software Real Time Analysis (RTA 1.13.48, HCS 1.5.15.1) and followed by generation of FASTQ sequence files by CASAVA, yielding a total of 1.66 Tbp (108.1 ± 2.8 million read pairs and 21.8 ± 0.6 Gbp per sample; mean ± standard deviation). Fastq files with the clean reads for all 76 samples are available at ENA under the BioProject accession number PRJEB52452^28^ (Supplementary Table 1).

### Bioinformatics workflow

**Fig. 2:**
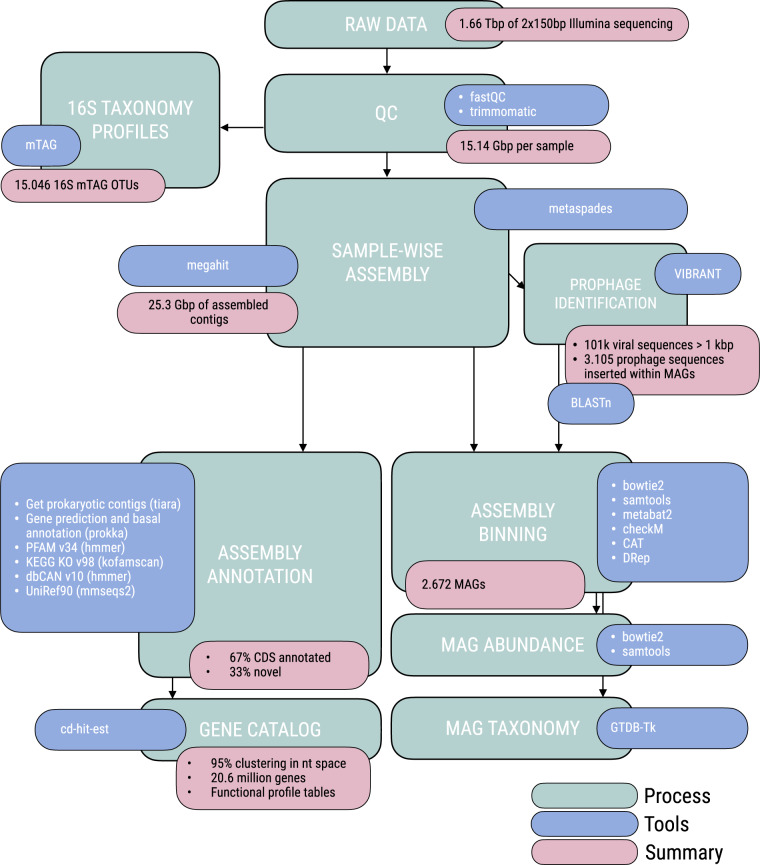
Bioinformatics workflow for processing metagenomes. Summary of the bioinformatics workflow used to process 76 metagenomes from 11 vertical profiles from the Malaspina Expedition, including seven depths from the surface to the ocean bottom or 4,000 m deep per sample. Processes or analyses are highlighted in green, the tools used in each process are highlighted in purple and selected results of each analysis are shaded in pink.

The bioinformatics workflow applied to this data set is summarized in Fig. 2 and consisted in the following steps:

The quality of raw read pairs was checked with FastQC v0.11.7 (https://www.bioinformatics.babraham.ac.uk/projects/fastqc/), and Illumina TruSeq adapter contamination was removed in Trimmomatic v0.38^29^ keeping adapter-free read pairs with contiguous quality over 20 and a minimum length of 45 bp with options “*ILLUMINACLIP:2:30:10 LEADING:3 SLIDINGWINDOW:4:20 MINLEN:45*”. Unpaired reads were discarded for further steps. After trimming, the dataset consisted of a total of 1.15 Tbp, with 81.5 ± 4.6 million clean read pairs and 15.1 ± 1.1 Gbp per sample (Supplementary Table 3).

Clean reads of each sample were assembled in Megahit v1.1.3^30^ with options “*--presets meta-large --min-contig-len 500*” to produce a total of 25.3 Gbp of metagenomic assemblies (332.9 Mbp ± 50.3, n = 76). The minimum contig size was set to 500 bp following *Tara* Ocean’s assembly protocol^31^ to make both datasets more homogeneous. In order to work only with the prokaryotic fraction of the assemblies, contigs were screened with Tiara v1.0.2^32^ with options “*--min_len 500 --pr*” and those marked as “eukarya” or “organelle” were not taken into account for further analyses. Eukaryotic contigs accounted for 11% of the total assembled basepairs (59.5 Mbp ± 28.3). Prokaryotic contigs were annotated in Prokka v1.14.6^33^ for gene prediction based in Prodigal^34^ (options *-c -m -g 11 -p meta*; considering only complete genes), clusters of orthologous groups (COGs)^16^, Enzyme Commission numbers (EC) and gene product name. Additionally, predicted genes amino acid sequences were annotated for protein families’ domains (PFAM v34)^17^ using HMMER v3.33 (*hmmsearch*)^35^ with option “*-E 0.1*”, the Kyoto Encyclopedia of Genes and Genomes Orthologs (KEGG KO)^18^ release v98.0 using KofamScan v1.3.0^36^ and options “*–format detail -E 0.01*”, and carbohydrate active enzymes (CAZy) using HMMER v3.33 (*hmmsearch*) against dbCAN v10^19^.

PFAM *hmmsearch* results were obtained with very low stringency (E = 0.1). The best hit was awarded for each model that aligned with no overlap to the predicted genes. This means that a single gene might have more than one protein domain annotation. When two or more hits were aligning in the same region, if the overlap was longer than half the length of the smaller alignment, the hit with larger bitscore was kept as the best one for that region.

Similarly, KofamScan (E = 0.01) results were filtered by keeping all hits with scores above the predefined thresholds for individual KOs (marked with an ‘*’), potentially assigning more than one KO to a single predicted gene.

Predicted coding sequences were taxonomically assigned by mapping them to UniRef90^20^, release 2021_03 from 9 of June 2021, with MMseqs2^37^ development version, commit 13-45111, with the taxonomy workflow options *“--max-accept 100 --tax-lineage 1 -e 1E-5 -v 3 -a*” and converted to table with *mmseqs createtsv*. All ranks out of domain, phylum, class, order, family, genus or species were removed from classification and missing fields were marked as “unclassified”. The lowest common ancestor for each sequence was also recorded. Genes from prokaryotic contigs classified as domain Eukarya were further removed from the dataset.

### Gene catalog

In order to reduce the redundancy of the predicted gene dataset, we clustered all coding DNA sequences longer than 100 bp to 95% nucleotide sequence similarity and 90% alignment coverage of the shorter sequence in CD-HIT v4.6.1^38^ with cd-hit-est and options “*-c 0.95 -G 0 -aS 0.9 -g 1 -r 1 -d 0 -s 0.8*”. We used the longest sequence of each cluster as the representative sequence, obtaining a catalog of 17,425,759 non-redundant genes. We refer to this set of coding sequences as the Malaspina Vertical Profiles Gene Database (M-GeneDB-VP)^23^. Functional and taxonomic annotation of the M-GeneDB-VP genes was inherited from the annotation of representative sequence of each cluster, as described above^23^.

### Functional profiling

**Fig. 3:**
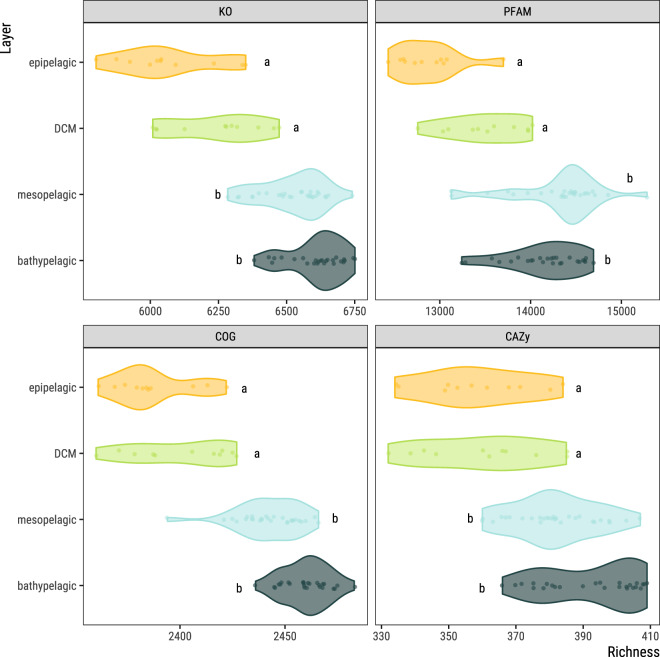
Prokaryotic functional richness (KO, PFAM, COG, CAZy) of 76 metagenomes grouped by depth layer. Functional richness of the prokaryotic fraction of 76 metagenomes from 11 vertical profiles from the Malaspina Expedition showed by ocean layer: epipelagic excluding the deep chlorophyll maximum (DCM) (from 0 to 200 m deep), DCM, mesopelagic (200 to 1,000 m deep) and bathypelagic (1,000 to 4,000 m deep), for KEGG orthologs (KO), protein families (PFAM), clusters of orthologous groups (COG) and carbohydrate-active enzymes (CAZy). Richness is calculated by converting gene abundances in the gene length normalized abundance tables for each feature to pseudo-counts and rarefying to 0.95 times the minimal sample sum with function *rtk* in R package rtk v0.2.6.1. Significant differences in richness values between ocean layers are depicted with different letters (Kruskal-Wallis, *p* < 0.05; Dunn’s post-hoc test with Holm correction for multiple comparisons).

Clean reads were back-mapped to the catalog with Bowtie2 v2.4.3^39^ and alignments were filtered with Samtools v1.15^40^ with option “*-F 4*” to keep only primary alignments. Reads mapping to catalog genes were counted in htseq-count from HTSeq v2.0.4^41^ with options “*--nonunique all* *--minaqual 0*” to build gene profiles per sample. As genes in a catalog are stripped from their genomic context, a read mapping to 2 contiguous genes in a genome would be randomly assigned to just one in the catalog. This option allows counting one read to more than one gene and to get a more inclusive representation of the abundance of each gene of the catalog by mapping it to all features it was assigned to, instead of randomly imputing it to only one. Counts were normalized by gene length in bp and then normalized by the geometric median abundance of 10 universal single-copy phylogenetic marker genes either for COGs (COG0012, COG0016, COG0018, COG0172, COG0215, COG0495, COG0525, COG0533, COG0541, and COG0552) or KOs (K01409, K01869, K01873, K01875, K01883, K01887, K01889, K03106, K03110, K06942) respectively. Normalizing coverage-corrected read counts by the abundance of these marker genes acts as a proxy to the number of gene copies per cell^42^. Functional profiles for COGs, PFAMs, KOs and CAZymes were calculated by adding up abundance values corresponding to genes annotated as a particular function, both from the gene length normalized table and the single-copy marker gene normalized table^23^. Functional richness was calculated by converting gene length normalized tables to pseudo-counts (multiplying abundance values by 10,000 and rounding to the next integer) and rarefying to 0.95 times the minimal sample sum with function *rtk* in R package rtk v0.2.6.1^43^ (Fig. 3).

**Fig. 4:**
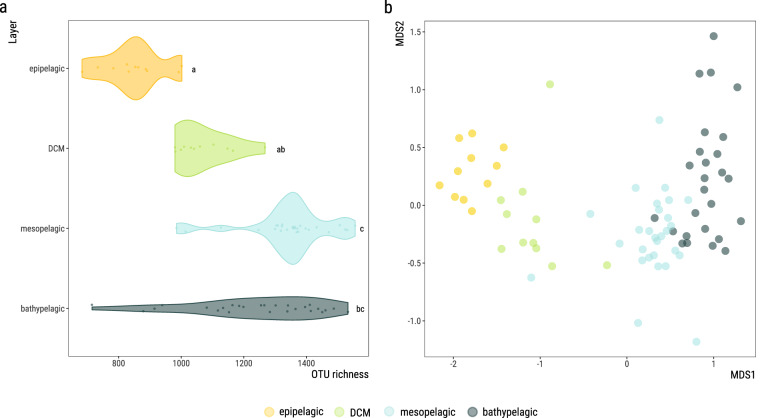
Sample prokaryotic taxonomic composition (richness and sample ordination) based in the analysis of mTAGS (16S rRNA SSU metagenomic fragments). (**a**) OTU richness based on 16S mTAGs analysis, showed by ocean layer: epipelagic excluding the deep chlorophyll maximum (DCM) (from 0 to 200 m deep), DCM, mesopelagic (200 to 1,000 m deep) and bathypelagic (1,000 to 4,000 m deep). Significant differences in richness values between ocean layers are depicted with different letters (Kruskal-Wallis, *p* < 0.05; Dunn’s post-hoc test with Holm correction for multiple comparisons). (**b**) Ordination plot (non-metric multidimensional scaling; Bray-Curtis distance) of 76 metagenomes from 11 vertical profiles based on their community composition (16S mTAG OTUs) colored by ocean layer as described above.

### Metagenome assembled genomes

**Fig. 5:**
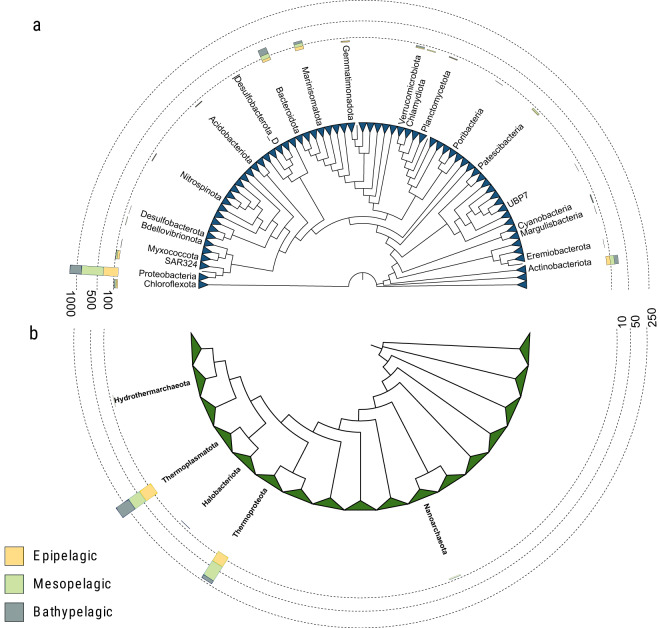
Diversity of prokaryotic MAGs from 76 metagenomes from 11 vertical profiles from the Malaspina Expedition based on GTDB-tk phylogenomic reconstruction and taxonomic classification. (**a**) Phylogenomic tree of Bacterial diversity. (**b**) Phylogenomic tree of Archaeal diversity. Stacked bar plots display the total number of MAGs obtained from each depth layer. All clades were collapsed at the level of Phylum and branch lengths were omitted to better display the tree topology.

Aiming to obtain high-quality Metagenome Assembled Genomes (MAGs), metagenomes were assembled individually using MetaSPAdes v3.13.0^44^. A Bowtie2 v2.3.4.1^39^ database was built using contigs longer than 2.5 kbp from all metagenomes. Next, post-QC metagenomic reads were queried against the aforementioned database using Bowtie2 in sensitive local mode. Output SAM files were converted to BAM and sorted using Samtools v1.15^40^. Sorted bam files were then used to calculate the contig abundance summary table using the *jgi_summarize_bam_contig_depths* script available through the Metabat repository (https://bitbucket.org/berkeleylab/metabat/src/master/). Finally, genome binning was performed for each individual metagenome using Metabat v2.12.1^45^. Completeness and contamination of the generated genome bins was estimated through CheckM v1.1.6^46^. Only bins with at least 50% completeness were kept for subsequent analysis. Among those, bins for which the contamination was estimated to be 5% or higher were subjected to a custom bin decontamination step as follows: first, each contig was assigned taxonomic annotation using CAT v5.2.3^47^, with option “*--fraction 0.05*”. Next, each contaminated bin was split into multiple sub-bins according to the class level taxonomic classification of each contig within it. The sub-bins were assessed for completeness and contamination as above. Finally, only bins with at least 50% completeness and less than 5% contamination were kept for subsequent analysis. These represent 2,672 medium and high-quality draft genomes according to MIMAG standards^48^ (Supplementary Table 5, BioStudies accession S-BSST1059^23^). Phylogenomic reconstruction and taxonomic classification of MAGs was carried out through GTDB-tk v1.7^49^ (Supplementary Table 5), and the resulting tree (Fig. 5) was decorated in iTOL^50^. MAGs were clustered using DRep v3.2.2^51^ into 1,228 non-redundant species clusters. Notably, 94 cluster representatives were obtained through our automated bin refinement method, meaning that without this step these 94 genome representatives would have yielded lower quality MAGs or not be identified in our dataset at all. This draws attention to the potential of automated bin refinement strategies as an efficient way to produce more MAGs and of higher quality, allowing for better characterization of genomic diversity within metagenomes. Taxonomic classification through phylogenomic reconstruction assigned MAGs to 22 bacterial and 5 archaeal phyla, which represented both ubiquitous and abundant taxa from marine ecosystems, such as Alphaproteobacteria, Cyanobacteria, and Thermoproteota, as well as low abundance taxa, such as Eremiobacterota, Margulisbacteria, Myxococcota, and UBP7. Overall, 509 out of 1,228 non-redundant MAGs were assigned to new species, one of which represented a new order within the class Planctomycetes.

### MAG relative abundances and community composition

**Fig. 6:**
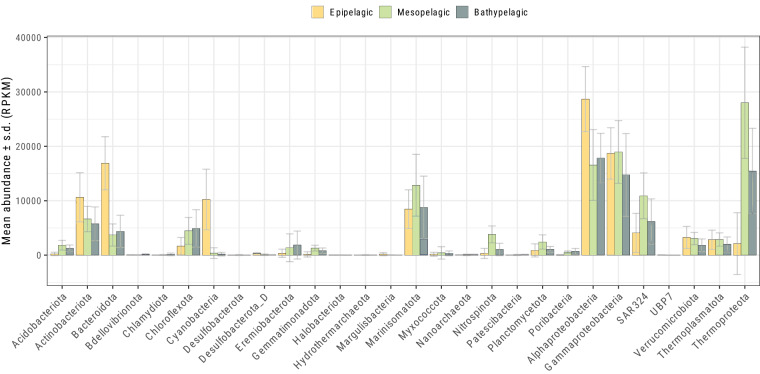
Taxonomic composition across communities from different depth zones, estimated by MAG relative abundances. Bar plots depict the mean relative abundances of MAGs grouped according to phyla (or class for Proteobacteria), across metagenomes grouped according to the depth zone from which metagenomes were obtained. Error bars represent standard deviations.

Relative abundances of MAGs and taxa were calculated as follows. A second Bowtie2 database was built, this time containing only the contigs associated with the set of 1,228 de-replicated medium and high-quality MAGs. Next, post-QC reads from the metagenomes were queried against the MDB using Bowtie2 in sensitive local mode. Output SAM files were converted to BAM and sorted using Samtools. Contig relative abundances were calculated as Reads Per Kilobase per Million total sequences (RPKM). MAG relative abundances, at each metagenome sample, were calculated as the sum of the RPKM values of the contigs according to the MAG to which they belonged. Finally, taxon relative abundances, at each metagenome sample, were calculated as the sum of the RPKM values of the MAGs according to the Phylum (or class for Proteobacteria) to which they were assigned (Fig. 6).

### Taxonomic profiling

Additional taxonomic profiling of the prokaryotic composition was added to the previous MAG community description. MAGs alone don’t use all the available sequencing information and rarer organisms may remain undetected. In order to capture as much taxonomic information of each metagenome and to be able to explore the community composition relationships between depth layers we identified and classified mTAGs, 16S ribosomal RNA gene small subunit (SSU) fragments, directly from the Illumina-sequenced metagenomes^21^ with mTAGs v1.0.4^52^, *profile* workflow with options “*-ma 1000 -mr 1000*”. This protocol is particularly suitable for metagenomes with short reads, as it takes advantage of a degenerated consensus reference database and an exhaustive search strategy, reducing the number of ambiguously mapped sequences that could not be used for classification. Briefly, the mTAGs pipeline extracts reads from the metagenome, which are identified as SSU-rRNA gene sequences by using hidden Markov models, and then maps them to a reference sequence database based on SILVA 138^53^, pre-clustered at 97% of sequence similarity and with degenerated consensus sequences within each OTU. It then classifies mTAGs conservatively to a taxonomic rank by considering its lowest common ancestor. Finally, it builds taxonomic profiles at different ranks, including the OTU level^23^. OTUs classified as “class Cyanobacteriia; order Chloroplast” or “family Mitochondria” were removed from the OTU counts table. The OTU count table was rarefied (5,861 reads/sample) using the *rrarefy* function in the R package vegan v2.5.7^54^ to correct for uneven sequencing depths among samples.

### Identification of viral sequences among assembled contigs

VIBRANT v1.2.1^55^ was used to identify viral genomic sequences derived from dsDNA viruses of archaea and bacteria among the assembled contigs. VirSorter2^55^ was applied to identify sequences derived from Nucleo-Cytoplasmic Large DNA Viruses (NCLDV), virophages (Lavidaviridae), and ssDNA viruses. Next, CheckV v0.8.1^56^ was applied to assess the quality (i.e., completeness and host contamination) of the obtained viral genomic sequences. A total of 123,976 viral genomic sequences were identified, among which 302 were considered high-quality (Completeness >  = 90%, and contamination = 0%) according to MIUVIG standards^57^. Computational host predictions were performed using PHIST version ed2a1e6^58^. For the PHIST analysis, only predictions with a maximum e-value of 2.384e-14 were considered, which yields approximately 85% class level prediction accuracy. The collection of 2,672 MAGs was used as a set of putative hosts. Putative hosts were assigned to 9,543 viral genomic sequences^23^, the most frequent host assignments were to Alphaproteobacteria (3,244 contigs), Gammaproteobacteria (1,826), Marinisomatota (894), Bacteroidota (881) and Actinobacteriota (734).

### Annotation of MAGs and viral genomic sequences

Coding DNA Sequences (CDS) derived from MAGs and viral genomic sequences^23^ were queried against three databases for annotation: (1) UniRef100^20^ using DIAMOND v2.0.7^59^, (2) KOFam^36^ using *hmmscan* in HMMER v3.3^35^, (3) PFAM^17^ using HMMER’s *hmmscan* as well. For all searches, only hits that displayed a bitscore ≥50 and e-value ≤ 10^−5^ were considered as valid hits and included in the annotation tables^23^.

## Data Records

All sequencing products described here, as well as the primary metagenome assemblies, can be found under BioProject accession number PRJEB52452 hosted by the European Nucleotide Archive^28^. ENA accession numbers for each metagenome sequencing run and for each megahit assembly are provided in Supplementary Tables 1, 4 respectively.

File 1: 17,425,759 non-redundant coding DNA sequences (gene catalog) can be found in MP-GeneDB-VP.fasta.gz^23^.

File 2: Prokka annotation for each CDS from the gene catalog, plus annotations for PFAM, KEGG-KO, CAZy and lowest common ancestor taxonomy can be found in file MP-GeneDB-VP-annotation-enhanced.tsv.gz^23^.

File 3: 16S rRNA mTAG-based OTU table of the 76 metagenomes can be found in file mp-mtags.otu.tsv^23^.

File 4: Counts of reads from each metagenome mapping to the gene catalog can be found in file MP-GeneDB-VP-raw-counts.tbl.gz^23^.

File 5: Counts of reads from each metagenome mapping to the gene catalog normalized by gene length can be found in file MP-GeneDB-VP-length-norm-counts.tbl.gz^23^.

File 6: Counts of reads from each metagenome mapping to the gene catalog annotated to COGs, normalized by gene length and 10 universal single copy COGs can be found in file MP-GeneDB-VP-length-norm-scgNorm-counts-cog.tbl.gz^23^.

File 7: Counts of reads from each metagenome mapping to the gene catalog annotated to KEGG KOs, normalized by gene length and 10 universal single copy KOs can be found in file MP-GeneDB-VP-length-norm-scgNorm-counts-ko.tbl.gz^23^.

File 8. Counts of reads from each metagenome mapping to the gene catalog normalized by gene length and aggregated per COG can be found in file MP-GeneDB-VP-length-norm-cog.tbl.gz^23^.

File 9. Counts of reads from each metagenome mapping to the gene catalog normalized by gene length and aggregated per KO can be found in file MP-GeneDB-VP-length-norm-ko.tbl.gz^23^.

File 10. Counts of reads from each metagenome mapping to the gene catalog normalized by gene length and aggregated per PFAM can be found in file MP-GeneDB-VP-length-norm-pfam.tbl.gz^23^.

File 11. Counts of reads from each metagenome mapping to the gene catalog normalized by gene length and aggregated per CAZy can be found in file MP-GeneDB-VP-length-norm-cazy.tbl.gz^23^.

File 12: fasta sequences for the 2,672 MAGs with estimated genome completeness above 50% and contamination below 5% can be found at file Malaspina-VP-MAGs.tar.gz^23^.

File 13. Functional annotation of each MAG can be found in file Malaspina-VP-MAGs_CDS-annotation.tsv.gz^23^.

File 14. Amino acid sequences of predicted genes in the MAGs sequences can be found in file Malaspina-VP-MAGs_CDS.faa.gz^23^.

File 15: Nucleotide sequences of predicted genes in the MAGs sequences can be found in file Malaspina-VP-MAGs_CDS.fna.gz^23^.

File 16: Viral genomic sequences can be found in file Malaspina_Profiles_Viruses_Genomic_Sequences.fasta.gz^23^.

File 17: Descriptive information on the viral genomic sequences can be found in file Malaspina_Profiles_Viruses_Genomic_Info.tsv^23^.

File 18: Virus-derived coding DNA sequences can be found in file Malaspina_Profiles_Viruses_CDS_Sequences.fna.gz^23^.

File 19: Information of the annotation of the protein encoding genes predicted in the viral genomic sequences can be found in file Malaspina_Profiles_Viruses_PEG_Annotation_Info.tsv^23^.

Underway and meteorological data measured on board R/V Hesperides for all 7 legs of the Malaspina Expedition 2010 on board R/V Hespérides are available from the Marine Technology Unit (UTM, CSIC)^60–66^.

## Technical Validation

Extracted DNA was quantified using a Nanodrop ND-1000 spectrophotometer (NanoDrop Technologies Inc, Wilmington, DE, USA) and the Quant_iT dsDNA HS Assay Kit with a Qubit fluorometer (Life Technologies, Paisley, UK).

The sequencing error rate was calculated by the sequencing center using PhiX147 phage DNA spikes (0.4% ± 0.1).

## Usage Notes

The metagenomic sequence files deposited at the ENA described here are raw sequences and have not been pre-processed in any way. Before using this data set for re-analysis it is advised to screen sequencing files with current quality-control tools such as the ones used here.

### Supplementary information

Supplementary tables

## Data Availability

All the software used to process the data set presented here is publicly available and distributed by their developers. All versions have been specified in the main text, along with the options used when departing from defaults. Custom scripts used in intermediate or summarizing steps are available at https://gitlab.com/malaspina-public/picoplankton-vertical-profiles. Code for bin decontamination step can be found at https://github.com/felipehcoutinho/QueroBins.
